# Dual Roles of Serine-Threonine Kinase Receptor-Associated Protein (STRAP) in Redox-Sensitive Signaling Pathways Related to Cancer Development

**DOI:** 10.1155/2018/5241524

**Published:** 2018-04-19

**Authors:** Ravi Manoharan, Hyun-A Seong, Hyunjung Ha

**Affiliations:** ^1^Department of Biochemistry, University of Madras, Guindy Campus, Chennai 600025, India; ^2^Department of Biochemistry, School of Biological Sciences, Chungbuk National University, Cheongju 28644, Republic of Korea

## Abstract

Serine-threonine kinase receptor-associated protein (STRAP) is a transforming growth factor *β* (TGF-*β*) receptor-interacting protein that has been implicated in both cell proliferation and cell death in response to various stresses. However, the precise roles of STRAP in these cellular processes are still unclear. The mechanisms by which STRAP controls both cell proliferation and cell death are now beginning to be unraveled. In addition to its biological roles, this review also focuses on the dual functions of STRAP in cancers displaying redox dysregulation, where it can behave as a tumor suppressor or an oncogene (i.e., it can either inhibit or promote tumor formation), depending on the cellular context. Further studies are needed to define the functions of STRAP and the redox-sensitive intracellular signaling pathways that enhance either cell proliferation or cell death in human cancer tissues, which may help in the development of effective treatments for cancer.

## 1. Introduction

Reactive oxygen species (ROS) mediate redox signaling critical for numerous cellular functions [1]. In general, moderate levels of ROS function as signals to activate stress-responsive survival pathways [2]. By contrast, high levels of ROS in cells and tissues can induce cell damage and activate cell death pathways. Therefore, maintenance of moderate levels of ROS in cells is important to support essential signaling pathways without causing cellular damage and death. Under the normal physiological conditions, the redox balance maintains the proper function of redox-sensitive signaling proteins and ensures that cells respond properly to endogenous and exogenous stimuli [3]. However, once redox state is unbalanced, oxidative stress is induced and tumor formation is promoted by initiating an aberrant induction of tumor progression signaling and disruption of cell death signaling [4]. Notably, ROS-sensitive signaling pathways that participate in cell proliferation, differentiation, and cell survival process are frequently elevated in many types of cancers [5].

Cell proliferation and cell death must be regulated to maintain tissue homeostasis in multicellular organisms. This regulation is achieved, in part, through many redox-sensitive intracellular signaling pathways that coordinate the processes of cell proliferation and cell death. These intracellular signaling events are influenced by protein-protein interactions, which involve various signaling molecules and protein modifications, such as protein phosphorylation that either activates or inactivates a target protein to perform a certain function.

Serine-threonine kinase receptor-associated protein (STRAP) was initially identified as a transforming growth factor *β* (TGF-*β*) receptor-interacting protein that inhibited TGF-*β* signaling, probably by stabilizing the association between TGF-*β* receptors and Smad7 and preventing the binding of Smad2 and Smad3 to TGF-*β* receptors [6]. STRAP localizes in both the cytoplasm and nucleus [7]. STRAP-deficient mouse embryonic fibroblasts (STRAP^−/−^ MEFs) showed higher levels of TGF-*β*-mediated transcriptional activation and growth inhibition than their wild-type counterparts [8]. TGF-*β* signaling is initiated by the binding of ligands, such as TGF-*β*1, to type I and II TGF-*β* receptors on the cell surface. Activated TGF-*β* receptors directly induce the downstream phosphorylation of the transcription factors Smad2 and Smad3, which undergo rapid homotrimerization and conversion to Co-Smad heterooligomers that contain Smad4. The heteromeric Smad complex then translocates into the nucleus, where it cooperates with other nuclear cofactors to regulate the transcription of target genes [9, 10].

**Figure 1:**
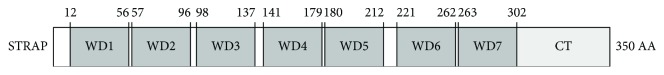
Domain structures of STRAP protein. STRAP protein contains seven WD40 repeats (WD1 to 7) and the C-terminal (CT) domain. Numbers indicate the amino acid residues corresponding to the domain boundaries.

The STRAP gene was first cloned from mouse embryonic [6] and human HepG2 [11] cDNA libraries. The human and mouse STRAP gene encodes a protein of 350 amino acids with a predicted molecular mass of 38 kDa. The full-length STRAP protein includes seven WD40 repeats (WD1 to 7) and a C-terminal (CT) domain (Figure 1). A WD40 repeat is composed of approximately 40 amino acids, often terminating in a tryptophan-aspartic acid (WD) dipeptide [12]. Equally important, WD40 repeats are frequently involved in protein-protein interactions [13]. In fact, the WD40 repeats within STRAP bind to various target proteins, thereby regulating cell proliferation and cell death [14–18]. Moreover, STRAP knockout mice showed embryonic lethality between embryonic days E 10.5 and E 12.5 due to defects in angiogenesis, cardiogenesis, somatogenesis, and neural tube closure, indicating that STRAP is required for normal embryo development [19]. Furthermore, the deregulation of STRAP has been implicated in tumorigenesis [8, 11, 18].

**Figure 2:**
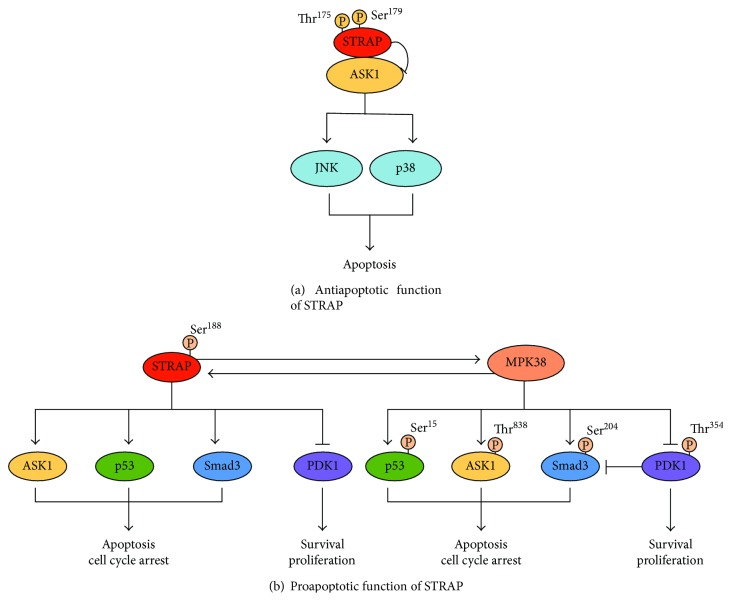
Differential regulation of STRAP functions by phosphorylation. **(**a) STRAP phosphorylation at Thr^175^ and Ser^179^ by ASK1 for STRAP-mediated inhibition of ASK1-induced cell death [16]. (b) STRAP Ser^188^ phosphorylation by MPK38 leads to cell death by modulating both STRAP- and MPK38-mediated ASK1, TGF-*β*, p53, and PI3K/PDK1 signaling pathways [14]. P: phosphorylated; ASK1: apoptosis signal-regulating kinase 1; PDK1: 3-phosphoinositide-dependent protein kinase-1; MPK38: murine protein serine/threonine kinase 38.

Posttranslational modifications (PTMs), such as protein phosphorylation, are critical for STRAP to exert its biological functions (Figure 2). STRAP stability and activity is mainly regulated by phosphorylation at threonine and serine residues. For instance, phosphorylation of STRAP at Thr^175^ and Ser^179^ by ASK1 is required for the negative regulation of ASK1 activity [16]. Murine protein serine/threonine kinase 38 (MPK38)-mediated of STRAP at Ser^188^ enhanced STRAP stability, thereby inducing the proapoptotic activity of STRAP via redox-sensitive ASK1, TGF-*β*, p53, and phosphoinositide 3-kinase (PI3K)/3-phosphoinositide-dependent protein kinase-1 (PDK1) signaling [14]. These data indicate that the phosphorylation of STRAP at specific residues plays an important role in determining STRAP-mediated cell proliferation and cell death, although further studies are needed to identify the other phosphorylation sites on STRAP.

Intense research in recent years has revealed that STRAP interacts with many redox-sensitive signaling proteins that regulate various cellular processes such as the cell cycle, proliferation, differentiation, survival, and apoptosis. Under the normal physiological conditions, STRAP promotes cell survival and proliferation and inhibits cell cycle arrest and apoptosis in normal and cancer cells, indicating that STRAP may function as an antiapoptotic protein [8, 16, 20]. By contrast, changes in redox balance induce cell death by STRAP in normal and cancer cells, implying that STRAP may also function as a proapoptotic protein [14, 21]. In this review, we summarize recent progress in understanding the potential cellular and molecular mechanisms of STRAP involved in cell proliferation, survival, and death. We also review the dual roles of STRAP in cancer, which may contribute to the development of a novel therapeutic option for cancer treatment.

## 2. Regulation of STRAP Activity by Protein-Protein Interactions

### 2.1. Antiapoptotic Activity of STRAP in Cells

The regulation of cell proliferation in multicellular organisms is a complex process, which is achieved, in part, by crosstalk between cell cycle progression and programmed cell death. STRAP promotes cell proliferation, which may be achieved through the inhibition of apoptosis and the activation of cell growth pathways. Furthermore, numerous STRAP-interacting proteins can positively or negatively regulate STRAP function. The following sections highlight our current understanding of the diverse regulatory mechanisms of STRAP in cell proliferation.

#### 2.1.1. STRAP*-*Mediated Inhibition of TGF*-β* Signaling Pathway

Nm23-H1, a tumor suppressor, enhances the STRAP-induced inhibition of TGF-*β* signaling via a redox-dependent interaction with STRAP [15]. The association of Nm23-H1 with STRAP is mediated by cysteine residues present in each of these two proteins, Cys^145^ in Nm23-H1 and Cys^152^ and Cys^270^ in STRAP. Consistently, this association was dependent on the presence of dithiothreitol or *β*-mercaptoethanol but not H_2_O_2_. The coexpression of Nm23-H1 with STRAP promotes the inhibition of TGF-*β*-induced apoptosis and promotes cell growth by STRAP. Another study suggests that PDK1 also regulates the STRAP-induced inhibition of TGF-*β* signaling [17]. Notably, PDK1 potentiates the STRAP-induced inhibition of TGF-*β* signaling by stabilizing the association between Smad7 and TGF-*β* receptors and preventing the nuclear translocation of Smad3. In addition to Nm23-H1 and PDK1, another STRAP-interacting protein, B-myb, was found to promote the STRAP-induced inhibition of TGF-*β* signaling. An amino-terminal DNA-binding domain and a region (amino acids 373–468) between the acidic and conserved regions of B-myb mediate the B-myb-STRAP interaction. This binding enhances the STRAP-mediated inhibition of TGF-*β* signaling by modulating complex formation between TGF-*β* receptors and Smad3 or Smad7. Furthermore, B-myb prevents the translocation of Smad3 in response to TGF-*β*1 [22]. Collectively, these findings add to the growing evidence that STRAP participates in the negative regulation of TGF-*β* signaling and subsequently promotes cell growth by directly interacting with many intracellular interacting partners such as Nm23-H1, PDK1, and B-myb.

#### 2.1.2. STRAP-Mediated Inhibition of ASK1 Signaling Pathway

Apart from its role in the TGF-*β* pathway, as discussed above, STRAP is involved in the suppression of cell death through a redox-dependent interaction with apoptosis signal-regulating kinase 1 (ASK1) [16]. ASK1, a member of the mitogen-activated protein kinase kinase kinase family, is activated by diverse stimuli, including ROS, tumor necrosis factor *α* (TNF-*α*), Fas, H_2_O_2_, and DNA damage. The activation of ASK1 leads to the stimulation of the c-Jun NH_2_-terminal kinase (JNK)/p38 signaling pathway, which is essential for cell death [23–25]. ASK1 has emerged as a key regulator of apoptosis, and its inactivation may directly contribute to the promotion of cell growth. We recently reported that STRAP interacts with ASK1 and subsequently inhibits ASK1 activity. The redox-dependent interaction of ASK1 and STRAP was mediated by cysteine residues present in each of these two proteins, Cys^1351^ and Cys^1360^ in ASK1 and Cys^152^ and Cys^270^ in STRAP. However, this interaction was disrupted by exogenous stimuli, including H_2_O_2,_ TNF-*α*, endoplasmic reticulum-induced stress (thapsigargin), and calcium overload (ionomycin). Furthermore, ASK1 phosphorylates STRAP at Thr^175^ and Ser^179^. Phosphorylation at these residues is important for stabilizing complex formation between ASK1 and its negative regulators, thioredoxin and 14-3-3 and/or preventing complex formation between ASK1 and its substrate mitogen-activated protein kinase kinase 3 (MKK3), thereby resulting in the inhibition of ASK1 signaling that regulates JNK and p38 kinases [16]. Consistently, STRAP suppressed H_2_O_2_-mediated apoptosis in an ASK1 phosphorylation-dependent manner by inhibiting ASK1 activity via direct protein-protein interactions (Figure 2(a)).

#### 2.1.3. STRAP-Mediated Stimulation of PDK1 Signaling Pathway

PDK1 is a serine-threonine kinase that phosphorylates and activates various downstream targets, including protein kinase C, S6 ribosomal kinase (S6K), glucocorticoid-induced kinase, and AKT1, to induce various cellular responses such as cell survival, proliferation, and differentiation [26–30]. STRAP was also identified as a binding partner of PDK1, an interaction requiring the catalytic domain of PDK1. The interaction between PDK1 and STRAP was increased by insulin treatment but decreased by TGF-*β*1 treatment. Moreover, STRAP positively regulates the PI3K/PDK1-mediated protection against TNF-*α*-induced apoptosis, thereby enhancing cell survival [17].

#### 2.1.4. STRAP-Mediated Stimulation of Nm23-H1-Induced Cell Growth

STRAP also regulates cell proliferation by modulating Nm23-H1-mediated signaling. Nm23-H1 was initially identified as a suppressor of metastasis due to its low expression in highly metastatic cell lines and tumors [31]. In addition to its role as a metastatic suppressor, Nm23-H1 also contributes to the proliferation and differentiation of cervical cancer and breast carcinoma cell lines and to the progression of the disease [32, 33]. However, the mechanism by which Nm23-H1 affects cell proliferation is unknown. We recently reported that STRAP interacts with Nm23-H1 in a redox-dependent manner and promotes cell growth. In HaCaT cells, a spontaneously transformed aneuploid immortal keratinocyte cell line, the overexpression of STRAP, but not a STRAP (C152S/C270S) cysteine mutant defective in binding to Nm23-H1, resulted in the promotion of Nm23-H1-induced cell growth [15]. These results indicate the possible involvement of STRAP in Nm23-H1-induced cell growth.

**Figure 3:**
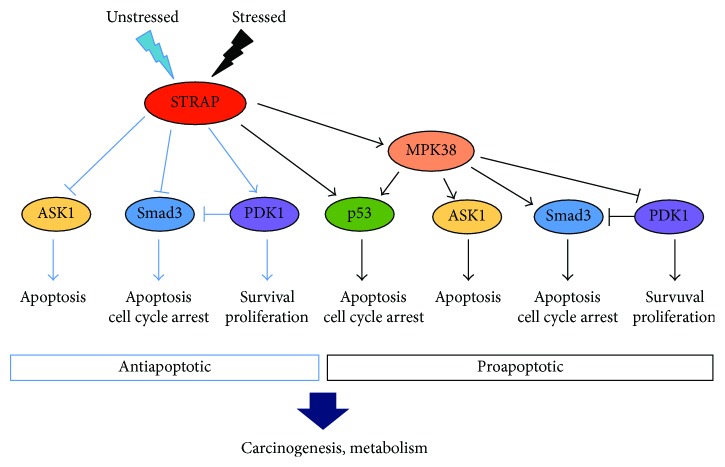
Main mechanisms through which STRAP exerts its double-faced role. Under the normal physiological condition, STRAP promotes cell survival and proliferation and inhibits cell cycle arrest and apoptosis, partly by regulating a number of signaling molecules, such as ASK1, Smad3, and PDK1. Upon treatment of cells with ASK1/TGF-*β*/p53 stimuli, STRAP promotes cell cycle arrest and apoptosis, partly by regulating p53 and MPK38 signaling molecules [14–17, 21, 38–41].

In summary, these findings reveal that STRAP modulates multiple mechanisms that contribute to the reduced sensitivity of cells to apoptosis, thereby promoting cell survival. STRAP is a key signaling molecule that regulates cell proliferation by controlling a broad range of biological processes such as cell growth, cell survival, cell cycle arrest, and apoptosis (Figure 3). The functions of STRAP are achieved through its large number of interacting partners and multiple modes of regulation. Therefore, an important future direction is to identify and characterize additional STRAP-interacting partners to better understand how the specific functions of STRAP are fulfilled through its interacting partners.

### 2.2. Proapoptotic Activity of STRAP in Cells

Although STRAP promotes cell survival and proliferation by inhibiting apoptosis, recent studies show that STRAP can also mediate cell death depending on the stimulus (Figure 3). Recently, this was supported by reports showing that STRAP associates with the p53 tumor suppressor and subsequently stimulates p53-mediated transcriptional activity [21, 22]. The p53 tumor suppressor is a transcriptional regulator; thus, it can regulate the expression of numerous target genes that induce cell cycle arrest, differentiation, and apoptosis in response to different cellular stresses [34]. p53 activity is modulated by various binding partners that induce the transactivation of target genes and different cellular outcomes [35]. Nm23-H1 and its binding partner STRAP associate with p53 and subsequently potentiate p53-mediated transcription. p53 activation by Nm23-H1 and STRAP is mediated by the removal of mouse double minute 2 homolog (Mdm2), a negative regulator of p53, from the p53-Mdm2 complex. Notably, both Nm23-H1 and STRAP directly interact with the central DNA-binding domain of p53 in a redox-dependent manner, thereby promoting its functions such as apoptosis and cell cycle arrest [21]. Additional studies suggest that the redox-dependent interaction of STRAP with MPK38 contributes to cell death through ASK1, TGF-*β*, p53, and PI3K/PDK1 signaling, leading to apoptotic cell death [14]; thus, these findings indicate that STRAP functions as a proapoptotic molecule.

## 3. Regulation of STRAP Activity by Phosphorylation

Recent studies report that the STRAP-mediated balance between apoptosis and cell proliferation is linked to phosphorylation events, and that STRAP phosphorylation at specific residues plays an important role in determining whether a cell proliferates or dies [14, 16]. MPK38, also known as maternal embryonic leucine zipper kinase, is a member of the AMP-activated protein kinase-related kinase family. It controls a variety of biological processes, including cell proliferation, survival, apoptosis, tumorigenesis, signal transduction, and metabolism [36, 37]. Specifically, MPK38 interacts with and phosphorylates diverse target proteins, thereby regulating their biological functions. For instance, MPK38 phosphorylates ASK1 at Thr^838^ and promotes ASK1-mediated apoptosis [38]. MPK38 also phosphorylates p53 at Ser^15^ [39] and Smad proteins (Ser^245^ of Smad2, Ser^204^ of Smad3, Ser^343^ of Smad4, and Thr^96^ of Smad7) [40], resulting in the stimulation of p53- and TGF-*β*-induced cell cycle arrest and apoptosis, respectively. In addition, MPK38 inhibits the activity of PDK1 via the phosphorylation of Thr^354^, which decreases cell survival [41].

### 3.1. MPK38 Regulates STRAP Activity through Ser^188^ Phosphorylation

Our recent study showed that MPK38 also interacts with STRAP to enhance its apoptotic functions. Phosphorylation of STRAP at Ser^188^ by MPK38 plays a central role in promoting the activation of MPK38-dependent ASK1, TGF-*β*, and p53 signaling and the inactivation of MPK38-dependent PI3K/PDK1 signaling, eventually leading to apoptotic cell death [14]. STRAP phosphorylation at Ser^188^ by MPK38 also affects complex formation between ASK1 and MKK3, TGF-*β* receptors and Smad3, p53 and Mdm2, and PDK1 and AKT1, which is critical for the activation of these signaling pathways [14]. Collectively, these studies provide strong evidence that STRAP can affect cell death via two mechanisms by directly regulating these signalings and indirectly regulating these signaling via MPK38 (Figure 2(b)). Although the above study was carried out in cultured cells, a study performed in mice demonstrated that the phosphorylation of STRAP at Ser^188^ also associates with cell proliferation and cell death through these signalings, resulting in apoptotic cell death [14].

### 3.2. ASK1 Regulates STRAP Activity through Thr^175^/Ser^179^ Phosphorylation

The phosphorylation of STRAP at specific residues dictates whether the protein will play a role in cell proliferation or cell death. Different upstream signals may regulate STRAP and activate different intracellular signaling pathways, thus leading to distinct cell functions. For example, STRAP phosphorylation at Thr^175^ and Ser^179^ by ASK1 promotes cell survival and cell proliferation by suppressing apoptosis [16], whereas increased stress induces cell death, which is mediated by MPK38 phosphorylation (Figure 2). Further studies are needed to understand the roles of STRAP in cell proliferation and cell death.

## 4. Roles of STRAP in Cancer

### 4.1. Tumor-Promoting Activity of STRAP in Tumor Development

#### 4.1.1. Inhibition of TGF-*β* Signaling Pathway by STRAP

In general, a balance among cell proliferation, survival, and apoptosis maintains cellular homeostasis [42]. Cancers can occur when this balance is disrupted, either by an increase in cell proliferation or a decrease in cell death [43]. Mounting evidence indicates that STRAP can promote tumor progression by inhibiting apoptosis and activating cell proliferation. Furthermore, STRAP protein expression is elevated in 60% of colorectal, 78% of lung, and 46% of breast carcinomas [8, 11, 44]. The overexpression of STRAP in different cell lines promotes cell proliferation and tumorigenicity in *in vitro* and *in vivo* experiments. For example, the knockdown of endogenous STRAP by STRAP-specific siRNAs decreases tumorigenicity, which clearly supports the role of STRAP in carcinogenesis [8]. Given that TGF-*β* acts as a tumor suppressor in normal cells, redox-sensitive TGF-*β* signaling exerts the tumor suppressive effects of TGF-*β*, thereby inhibiting tumor development [45]. Furthermore, the overexpression of STRAP inhibits TGF-*β*-mediated growth suppression by increasing Smad7 binding to TGF-*β* receptors, which induces cell proliferation and tumor development [7]. Studies in various cancer cell lines also demonstrate that STRAP augments cell proliferation and oncogenesis by blocking the antiproliferative effects mediated by TGF-*β* signaling. Equally important, STRAP^−/−^ MEFs show enhanced TGF-*β*-mediated transcriptional activation and growth inhibition. For example, STRAP overexpression alleviates the TGF-*β*-induced inhibition of cell growth and induces the tumorigenicity of lung adenocarcinoma (A549) and colon adenocarcinoma (FET) cells [8], indicating an important role of STRAP in tumor development through negative regulation of TGF-*β*-mediated growth suppression.

#### 4.1.2. Stimulation of Notch and Wnt/*β*-Catenin Signaling Pathways by STRAP

A recent study also implicates that STRAP has a role in the progression of colorectal cancers (CRCs). STRAP expression was elevated in all stages of colorectal cancer, and the tumor growth was inhibited in heterozygous STRAP knockout mice. Importantly, STRAP activated Notch signaling by inhibiting polycomb repressive complex 2 assembly, leading to colon carcinogenesis [46]. STRAP also promotes Wnt/*β*-catenin signaling, leading to the development and progression of CRC. Notably, STRAP binds to GSK-3*β* and stabilizes *β*-catenin by inhibiting its ubiquitin-dependent degradation, resulting in the stimulation of Wnt/*β*-catenin signaling in CRC cells. Consistent with this observation, the knockdown of STRAP in murine colon carcinoma cell lines inhibited tumorigenesis, invasion, and metastasis, demonstrating that STRAP increases the invasion and metastasis of CRC partly via the inhibition of the ubiquitin-dependent degradation of *β*-catenin and the enhancement of Wnt/*β*-catenin signaling [47].

#### 4.1.3. Downregulation of E-cadherin and p21^Cip1^ Promoter Activities by STRAP

Analysis of clinical data from the cancer genome atlas reveals that the level of STRAP mRNA expression is upregulated in lung adenocarcinoma with metastasis, strongly implying that STRAP participates in the pathology of lung adenocarcinoma metastasis [48]. In addition, STRAP inhibits Sp1-dependent transcription, resulting in the downregulation of the tumor suppressor genes E-cadherin and p21^Cip1^, thereby promoting tumor progression in non-small-cell lung cancers. Therefore, the increased expression of STRAP in lung cancer contributes to the downregulation of E-cadherin and p21^Cip1^, which in turn leads to tumor progression [49].

#### 4.1.4. Enhancement of PDK1 Signaling Pathway by STRAP

Apoptosis, the major form of cellular suicide, is central to various physiological processes, including the maintenance of homeostasis in multicellular organisms. The inhibition of apoptosis can activate cell survival factors that facilitate the continuous proliferation in cancer cells [42]. STRAP promotes tumor progression through the inhibition of apoptosis and the activation of cell survival [15, 16]. The activation of PDK1 signaling has been implicated in cell proliferation, survival, and tumorigenesis [50]. PDK1 also inhibits TGF-*β*-mediated cell growth arrest and apoptosis by directly interacting with Smad proteins [51], revealing that PDK1 inhibition may be beneficial for tumor suppression. Thus, PDK1 inhibitors are currently being tested as anticancer drug*s* [52, 53]. STRAP also interacts with PDK1 and promotes the phosphorylation of PDK1 substrates, including S6K, AKT1, and Bad, leading to enhanced cell survival [17]. STRAP also indirectly inhibits cell cycle arrest and apoptosis by promoting the PDK1-mediated suppression of TGF-*β* signaling, resulting in enhanced cell survival [17]. These results clearly indicate that STRAP promotes cell survival and cell growth via PDK1, thereby contributing to tumor progression.

#### 4.1.5. Stimulation of Nm23-H1 Activity and Inhibition of ASK1 Activity by STRAP

STRAP was found to potentiate the Nm23-H1-induced growth of HaCaT cells through a redox-dependent interaction with Nm23-H1 [15]. Nm23-H1 was previously reported to affect proliferation and differentiation of cervical cancer and breast carcinoma cell lines [32, 33], suggesting that STRAP may also contribute to the progression of cervical and breast tumors. However, the exact nature of this mechanism is unknown, and further studies are needed to evaluate STRAP and Nm23-H1 activities during tumor progression. On the other hand, ASK1 functions as a tumor suppressor due to its ability to induce apoptosis of both breast cancer and lung adenocarcinoma cell lines [54, 55]. STRAP interacts with ASK1, which inhibits its kinase activity and the subsequent ASK1-induced apoptosis, thereby promoting cell survival and cell growth [16]. Overall, high level of STRAP expression in various cancers implies that STRAP has a role in tumor growth and aggressiveness, and therefore, inhibition of STRAP may be an attractive cancer therapeutic target.

### 4.2. Tumor Suppressive Activity of STRAP during Tumorigenesis

STRAP overexpression associates with the progression of many different types of tumors, although several investigators report contradictory results. For example, STRAP directly regulates the most important tumor suppressor, p53, in cervical cancer (HeLa), colorectal carcinoma (HCT116), and breast cancer (MCF7) cell lines. The overexpression of STRAP leads to increased p53-induced apoptosis and decreased cell proliferation, whereas the loss of STRAP has the opposite effects, indicating that STRAP plays a key role in tumor suppression [21, 22]. Given that p53 plays a critical role in numerous cellular processes, including cell cycle arrest, differentiation, apoptosis, and tumor suppression, the loss of p53 function is a common feature of human cancers [56]. Functionally, STRAP and its interacting partner Nm23-H1 directly bind to and stabilize p53 by dissociating Mdm2, resulting in the induction of p53-induced apoptosis and cell cycle arrest [21]. These results indicate that STRAP proteins are also responsible for tumor suppression.

Equally important, the PTM of STRAP by protein kinases, such as ASK1 and MPK38, plays an important role in determining the pro- or antiapoptotic function of STRAP. For example, STRAP phosphorylation at Thr^175^ and Ser^179^ by ASK1 is required for STRAP-mediated inhibition of ASK1-induced cell death [16]. By contrast, MPK38-mediated STRAP phosphorylation at Ser^188^contributes to the proapoptotic function of STRAP by modulating STRAP-dependent ASK1, TGF-*β*, p53, and PI3K/PDK1 signaling [14]. However, further studies are needed to clarify the mechanisms regulating STRAP function.

## 5. Conclusions

STRAP is an even more complex regulator of cellular functions than was previously thought, and its roles in the regulation of the redox-sensitive TGF-*β* cascade and the promotion of cell growth continue to be investigated. Apart from its role in the TGF-*β* cascade, additional details on the function and regulation of STRAP are rapidly emerging. For example, recent studies show that STRAP plays a critical role in the regulation of both cell proliferation and cell death in response to various stresses, accompanied by changes in the redox status, by interacting with multiple target proteins. Studies on STRAP-mediated redox signaling that promotes either cell proliferation or cell death, as well as studies on the significance of phosphorylation in these events, are at best preliminary. Therefore, further studies are needed to identify additional STRAP phosphorylation sites involved in the regulation of cell proliferation and cell death.

Although recent studies report conflicting results on the roles of STRAP in cancer, the tumor-promoting effects of STRAP, including the induction of cell proliferation and cell survival and the inhibition of apoptosis, were observed in numerous cancer cells. However, the tumor suppressive effects of STRAP have not yet been confirmed in cancer tissues, although the proapoptotic function of STRAP was observed in normal and cancer cells. Hence, further studies investigating the dual functions of STRAP, as well as its regulation by redox-dependent signaling, which induces either cell death or cell proliferation in human cancers, are needed because they may contribute to the development of more effective cancer treatments.
